# An Improved Metal-Packaged Strain Sensor Based on A Regenerated Fiber Bragg Grating in Hydrogen-Loaded Boron–Germanium Co-Doped Photosensitive Fiber for High-Temperature Applications

**DOI:** 10.3390/s17030431

**Published:** 2017-02-23

**Authors:** Yun Tu, Lin Ye, Shao-Ping Zhou, Shan-Tung Tu

**Affiliations:** 1 Key Laboratory of Pressure Systems and Safety (Ministry of Education), School of Mechanical and Power Engineering, East China University of Science and Technology, Shanghai 200237, China; ytu@ecust.edu.cn (Y.T.); shpzhou@ecust.edu.cn (S.-P.Z.); 2 Laboratory of Smart Materials and Structures, Centre for Advanced Materials Technology, School of Aerospace, Mechanical and Mechatronic Engineering, The University of Sydney, Sydney 2006, Australia; lin.ye@sydney.edu.au

**Keywords:** regenerated fiber Bragg grating (RFBG), metal-packaged, strain sensor, photosensitive fiber, high temperature, strength degradation, structural health monitoring

## Abstract

Local strain measurements are considered as an effective method for structural health monitoring of high-temperature components, which require accurate, reliable and durable sensors. To develop strain sensors that can be used in higher temperature environments, an improved metal-packaged strain sensor based on a regenerated fiber Bragg grating (RFBG) fabricated in hydrogen (H_2_)-loaded boron–germanium (B–Ge) co-doped photosensitive fiber is developed using the process of combining magnetron sputtering and electroplating, addressing the limitation of mechanical strength degradation of silica optical fibers after annealing at a high temperature for regeneration. The regeneration characteristics of the RFBGs and the strain characteristics of the sensor are evaluated. Numerical simulation of the sensor is conducted using a three-dimensional finite element model. Anomalous decay behavior of two regeneration regimes is observed for the FBGs written in H_2_-loaded B–Ge co-doped fiber. The strain sensor exhibits good linearity, stability and repeatability when exposed to constant high temperatures of up to 540 °C. A satisfactory agreement is obtained between the experimental and numerical results in strain sensitivity. The results demonstrate that the improved metal-packaged strain sensors based on RFBGs in H_2_-loaded B–Ge co-doped fiber provide great potential for high-temperature applications by addressing the issues of mechanical integrity and packaging.

## 1. Introduction

As a result of the global energy deficit and environmental deterioration, most plants tend to be larger scale in operations at higher operating parameters in order to improve energy conversion efficiency and productivity with reduced environmental impact. This could, unfortunately, lead to unexpected and fatal industrial accidents due to material deterioration at high temperatures leading significant loss in assets, and at times, even human life. It is most improbable that the integrity of a component before operation could be guaranteed merely using a conventional design due to the time-varying service conditions, particularly at a high temperature [1]. Structural health monitoring is thus an essential procedure to ensure the safety of the plant by providing real-time, reliable and accurate information about the condition of critical components. 

Since the local strain has been used as an indicator of the creep condition of high-temperature components, given the monotonic relationship between strain and creep life, strain measurements have been considered as the most reliable method for structural health monitoring of these components. However, traditional strain gauges are not reliable for prolonged measurements at high temperatures [2], in addition to nonlinearity of thermal-induced apparent strains and susceptibility to electromagnetic interference (EMI). Measurement accuracy with optical strain gauges and digital image correlation (DIC) is also adversely affected by insufficient image quality due to the inevitable degradation of markers exposed to high temperatures with time [3]. Therefore, strain measurements at high temperatures, where it is difficult to guarantee reliability and durability of the sensors, have been a long-standing challenge, with numerous potential industrial applications over decades, e.g., in the processing, energy and aerospace industry. 

Optical fiber sensors (OFSs) are well suited to structural health monitoring due to a number of advantages over their electrical counterparts, such as small size, light weight, electrically passive operation, high sensitivity, and resistance to electromagnetic interference and corrosion. In particular, wavelength encoded fiber Bragg grating (FBG) sensors have inherent self-referencing and wavelength multiplexing capabilities that allow them to be easily serialized in a single optical fiber and spliced to telecommunication fibers for remote, distributed and multi-parameter sensing [4]. However, conventional type-I FBGs exhibiting a strong decay at high temperatures can only operate in principle up to 300 °C for lengthy periods [5]. Many of research efforts have been expended towards the investigation of thermally stable gratings at high temperatures, including formation of type-I*n* (type-IIA) gratings [6,7] and type-II gratings [8], writing by femtosecond lasers [9], formation of surface relief FBGs [10], and tailoring of glass composition [11,12,13]. In the past decade, another variant, regenerated fiber Bragg gratings (RFBGs), with superior high-temperature stability has been found and considered as the essential potential for high-temperature applications [14,15].

As fiber-optic strain sensors must monitor strains and ensure long-term reliability in adverse environmental conditions, appropriate sensor packaging and attachment are crucial for their operation and lifetime. A common solution is to encapsulate an optical fiber sensor with an epoxy, which is indeed a simple method at room temperature, but most epoxies degrade after exposure to high temperatures over 400 °C. To overcome the limitation of this conventional encapsulation method, an all-metal packaging process has been proposed for protecting the bare RFBGs from environmental attack as well as easily attaching the RFBG-based strain sensors to metallic high-temperature components [16,17]. The process generally includes two steps. In the first step, one- or two-layer metallic films are typically deposited on the bare RFBGs as an adhesive and/or conductive layer by low temperature processes such as electroless plating [16,18], physical vapor deposition (e.g., magnetron sputtering [17,19] and evaporation deposition [20]), and laser-assisted maskless micro-deposition [21]. This occurs in order to achieve reliable bonding between glass and metal, in addition to allowing to electroplate nickel coating on the metallic films as a protective layer. After that, the metal-coated RFBGs are embedded into the metallic substrate of a high melting temperature by using brazing [16], ultrasonic consolidation [22], laser additive manufacturing [19,23], or nickel electroplating [17,21]. Careful selection of the packaging materials and processes is critical to ensure the long-term survivability of the package itself under high temperatures, and to achieve a strong and reliable glass-to-metal bond without mechanical or thermal damage. 

Besides the packaging issue, the mechanical integrity of optical fibers after annealing at a high temperature for regeneration is another vital issue which impedes the practical utility of the RFBG-based strain sensors, given that the silica fibers are found to become mechanically clearly weaker upon annealing, as reported by the authors and other researchers [24,25,26,27]. It is observed that cooling to room temperature after heating the silica fibers to a high temperature induces an additional significant reduction in mechanical strength compared to those tested at this high temperature at which annealing occurs. The degree of weakening becomes more severe with an increased temperature of annealing. Accordingly, the metal-packaged strain sensors based on the use of the RFBGs fabricated in standard telecommunication silica fibers (Corning Inc., SMF-28, Corning, NY, USA) can only be used up to 400 °C [17], as the mechanical strength of the standard SMF-28 silica optical fibers in which the RFBGs require the regeneration temperature of around 900 °C was observed to considerably degrade after annealing at 900 °C [26]. However, annealing at a high temperature is necessary for the fabrication of the RFBGs, although it usually leads to mechanical strength degradation of the silica optical fibers. The annealing temperature thus needs to be carefully chosen as a function of the anticipated operating temperature and duration in order to extend the application of RFBG to high-temperature conditions.

In this paper, the objective is to develop an RFBG-based strain sensor capable of application in higher temperature environments, e.g., fossil-fuel power plants operating at temperatures up to 540 °C. A metal-packaged strain sensor prototype is developed using an RFBG fabricated in hydrogen (H_2_)-loaded boron–germanium (B–Ge) co-doped photosensitive fiber (Fibercore Ltd., PS1250/1500, Southampton, UK). The PS1250/1500 fiber, in which the RFBGs require a relatively lower regeneration temperature (of 500 °C) will lead not only to better mechanical integrity but also to better high-temperature stability than the SMF-28 fiber that was used in fabricating the high-temperature strain sensors [17]. Thus, the RFBGs in PS1250/1500 fiber are chosen as base-sensing elements in the present work, to develop high-temperature strain sensors. The fabrication process of the strain sensor prototype is elaborated, and the sensor prototype and its corresponding bare RFBG are characterized at high temperatures under uniaxial tensile loading. Numerical simulation of the sensor is carried out based on the basic principle of strain measurements using FBGs and three-dimensional (3-D) finite element (FE) modeling to analyze the mechanical response of the metal-packaged strain sensor.

## 2. Strain Sensing Principles of Fiber Bragg Gratings

An FBG is formed as a periodic variation in the refractive index of the core of a photosensitive single-mode optical fiber. When a broadband light source is coupled to the optical fiber containing an FBG, the grating diffractive properties promote that only a very narrow wavelength band is back-reflected. Thus, the use of FBG sensors relies on the determination of the center wavelength of the back-reflected narrow band, called the Bragg wavelength, $\lambda_{B}$, defined by the Bragg condition [28]

(1) $$\lambda_{B}=2n_{\mathrm{eff}}\Lambda$$

where $n_{\mathrm{eff}}$ is the effective refractive index of the fiber core and Λ is the grating period. Both $n_{\mathrm{eff}}$ and Λ are affected by changes in deformation and temperature. Using Equation (1), the shift in the Bragg wavelength, $\Delta \lambda_{B}$, due to axial strain and temperature changes, is given by [29]:

(2) $$\Delta \lambda_{B}=2\left(\Lambda \frac{\partial n_{\mathrm{eff}}}{\partial l}+n_{eff}\frac{\partial \Lambda }{\partial l}\right)\Delta l+2\left(\Lambda \frac{\partial n_{\mathrm{eff}}}{\partial T}+n_{eff}\frac{\partial \Lambda }{\partial T}\right)\Delta T$$

where Δl and ΔT are the changes in grating length and temperature, respectively.

The first term in Equation (2) represents the strain effect on an optical fiber. When the fiber is only axially strained, the Bragg wavelength varies due to the changes in the grating period and the photoelastic-induced changes in the refractive index. In this case, the strain effect term in Equation (2) can be expressed as

(3) $$\Delta \lambda_{B}=\lambda_{B}\left\{\varepsilon_{z}-\frac{n_{\mathrm{eff}}^{2}}{2}\left[p_{12}\varepsilon_{z}+\left(p_{11}+p_{12}\right)\varepsilon_{r}\right]\right\}$$

where $p_{11}$ and $p_{12}$ are the components of the strain-optic tensor, and $\varepsilon_{z}$ and $\varepsilon_{r}$ are the axial and radial strains in the optical fiber respectively. Typical values for the strain-optic constants and effective refractive index for typical B–Ge co-doped photosensitive fibers are $p_{11}=0.113$, $p_{12}=0.252$ and $n_{\mathrm{eff}}=1.455$ [30]. For an FBG with Bragg wavelength of 1550 nm, the typical strain sensitivity is approximately a 1.2-pm change in Bragg wavelength as a result of applying a strain of 10^−6^ to the grating.

## 3. Metal-Packaged Strain Sensor Prototype

The study and development of the improved metal-packaged strain sensor prototype using an RFBG fabricated in H_2_-loaded PS1250/1500 photosensitive fiber as the sensing element are articulated in three aspects.

### 3.1. Fabrication of Strain Sensor Prototype

The fabrication of the metal-packaged RFBG strain sensor prototype using the metal-coated RFBG embedded in the steel substrate could be divided in to four steps:

*Step 1*: Customized type-I seed FBGs used in the present work were written in H_2_-loaded PS1250/1500 photosensitive fiber including 10 mol % of GeO_2_ and 14–18 mol % of B_2_O_3_ through a phase mask with a reflectivity of ~80%, a 3-dB reflection bandwidth of less than 0.3 nm and a grating length of 8 mm, which were produced by a commercial company.

**Figure 1:**
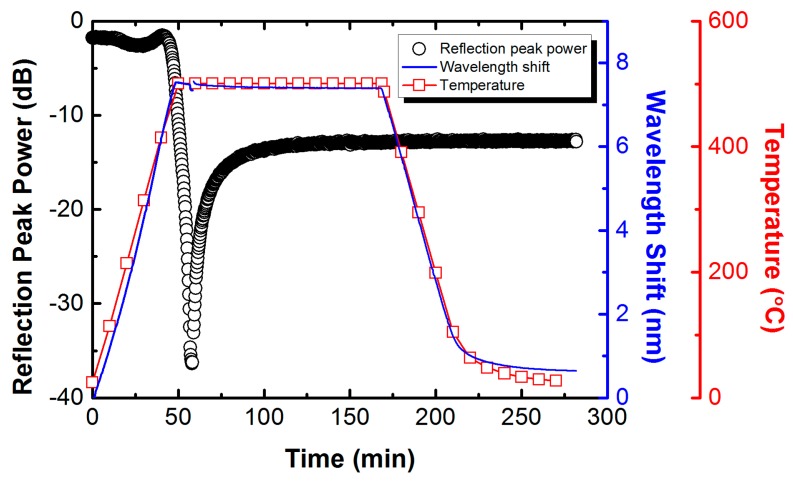
Typical evolution of the reflection peak power and the Bragg wavelength shift of one grating with the corresponding temperature profile during annealing for fabrication of the regenerated fiber Bragg grating (RFBG) in H_2_-loaded PS1250/1500 fiber.

*Step 2:* RFBGs were regenerated by annealing process. The type-I seed FBG was loosely placed in a capillary quartz tube (1 mm inside diameter and 2.5 mm outside diameter) horizontally inserted into the central area of a horizontal miniature tube furnace in order to maintain the temperature uniform along the grating and avoid any mechanical or thermal strain on the fiber. Then, a calibrated armored N-type thermocouple with a precision of ±0.5 °C was assembled together with the capillary quartz tube, and its tip was adjacent to the grating in order to control the temperature. It was built into the furnace’s feedback during the annealing throughout which the spectral behavior of the grating was monitored in reflection by a commercial FBG interrogator (Micron Optics, Inc., Sm125-500, Atlanta, GA, USA). The tabular furnace is custom-made with a length of 40 mm to anneal a short length of the fiber, as the mechanical strength of the fiber is considerably reduced by the annealing treatment and polymer coating is also removed via thermal stripping. Coating is essential to protect the fiber surface from handling damage during subsequent fabrication. The furnace is equipped with a quartz tube with an inside diameter of 8 mm and a length of 40 mm to homogenize the temperature inside the furnace. The furnace temperature was raised from room temperature to 500 °C within 50 min, and subsequently held constant at the temperature of 500 °C for ~120 min, which is the temperature triggering regeneration. To the best of the present authors’ knowledge, this temperature is the lowest regeneration temperature observed for the B–Ge co-doped photosensitive fiber, where a typical annealing process used for regeneration of RFBGs is shown in Figure 1. As the temperature approached 500 °C, the reflection peak power of the seed FBG started to decay drastically until it fell below the noise floor in a few minutes after the temperature reached 500 °C. After that, a regenerated grating appeared at longer wavelength after ~8 min at the temperature of 500 °C where the isothermal annealing was performed for ~120 min, followed by its reflection strength being increased gradually to a maximum until stability. No variation was observed in the strength of the RFBG as the temperature was decreased to room temperature over 90 min. The reflection spectra of the RFBG and its corresponding seed FBG was recorded at room temperature of 21 °C by the FBG interrogator.

*Step 3:* The multilayer metal-coated optical fiber containing the RFBG was fabricated by depositing a titanium (Ti) film on the fiber as an adhesive layer followed by a silver (Ag) film on the adhesive layer as a conductive layer by magnetron sputtering, and subsequently a nickel (Ni) coating on the conductive layer as a protective layer by electroplating. The details of this step have been described in our previous work [31].

*Step 4:* A series of experiments was carried out for successfully packaging the multilayer metal-coated RFBG into a P91 steel substrate to obtain a metal-packaged strain sensor prototype by using the RFBG fabricated in H_2_-loaded PS1250/1500 photosensitive fiber by means of the same process of all-metal packaging used for the RFBG fabricated in standard SMF-28 telecommunication fiber described in our previous work [17].

### 3.2. Characterization of Strain Sensor Prototype

**Figure 2:**
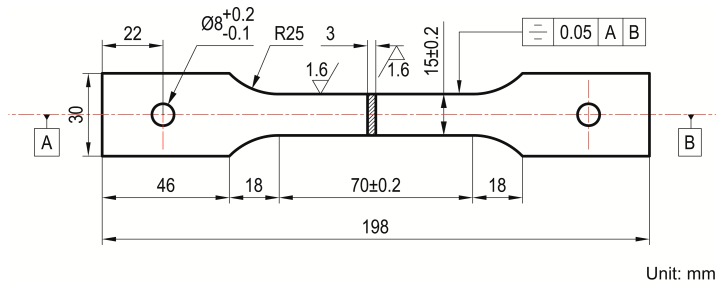
Pin-loaded sheet tensile test specimen with 70-mm gauge length.

For the determination of strain characteristics of metal-packaged RFBG strain sensors, uniaxial tensile tests were carried out on a P91 steel sheet tensile test specimen with the attached sensor prototype. The specimen has a gauge length of 70 mm with other dimensions shown in Figure 2. The sensor was mounted at the center of the gauge section along the center line using electrical resistance spot welding at both ends, minimizing adverse thermal effects on the specimen. An electromechanical universal test machine (MTS-SANS, CMT5504) instrumented with a furnace was used to produce strains in the test specimen by applying tensile loading, as described in detail in our previous work [17]. The specimen was pinned to grips, taking care to avoid bending or torsion loading to the sensor pigtail. The sensor was connected to the Sm125-500 FBG interrogator to assess the Bragg peak in the reflection spectrum measured during the mounting procedure. After that, the temperature was increased at a uniform rate to the desired temperatures of 100 °C, 200 °C, 300 °C, 400 °C, 500 °C and 540 °C, and kept constant for around 20 min before tensile testing. In order to maintain a nearly uniform temperature over the gauge length of the specimen, the temperature gradient defined by the difference in three calibrated N-type thermocouples was measured, with one mounted at the opposite side of the sensor and the other two mounted near two clamping fixtures. The test temperature was controlled within a tolerance range of ±2 °C and the temperature differences in the three points did not exceed 3 °C during tensile testing. The tensile force was applied to the specimen with a load interval of 1 kN or 0.5 kN and held constant at each load level for 2 min to obtain multiple measurements for defining the average value. The maximum tensile force did not exceed 8.0, 8.0, 8.0, 7.0, 6.5, 6.0, and 5.5 kN at the corresponding test temperatures of room temperature (26.5 °C), 100 °C, 200 °C, 300 °C, 400 °C, 500 °C and 540 °C, to avoid undesirable plastic deformation of the P91 steel specimen. 

In order to compare the strain characteristics of bare and packaged RFBG sensors, uniaxial tensile tests were also performed on the bare RFBG by using the test apparatus described in our previous work [17]. The fiber containing the RFBG was carefully wrapped around the two capstans and its ends were mechanically griped. The fiber was heated to the temperatures of room temperature (21 °C), 100 °C, 200 °C, 300 °C, 400 °C, 500 °C and 600 °C and kept at each temperature for at least 20 min before being tested. During the test, the fiber was stretched at a load interval of 0.2 N and kept constant at each load level for 2 min during which the temperature was held constant to obtain multiple measurements for defining the average value. The maximum force of 4 N, corresponding to approximately 0.4% strain, was applied to the fiber containing the RFBG to avoid the rupture of the fiber, considering the fracture force of silica optical fiber after annealing at a temperature of 500 °C not exceeding 8 N as discussed in our previous work [26].

The load and the corresponding Bragg wavelength were recorded simultaneously from the load cell and the FBG interrogator during tensile tests of both the P91 steel specimen with the RFBG sensor and the bare fiber with the RFBG.

### 3.3. Numerical Modelling

**Figure 3:**
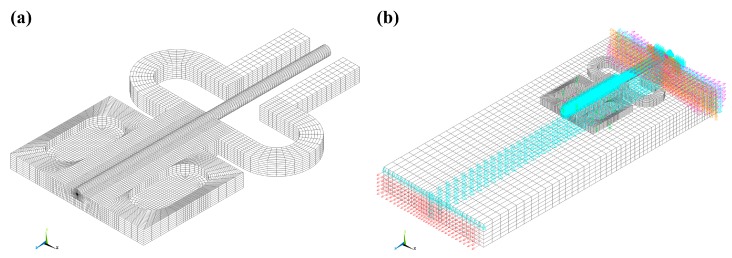
Three-dimensional (3-D) finite element (FE) model: (**a**) one half of a meshed model of a metal-packaged RFBG strain sensor, and (**b**) boundary conditions and applied load.

The 3-D FE modeling approach, which has been proposed and detailed in our previous work [17], was applied to modeling of the metal-package RFBG strain sensor prototype in the present work in order to find the state of stress and strain in the embedded optical fiber. The mechanical properties of the materials used in the 3-D FE analysis are listed in Table 1. One half of the structural model of a specimen with a metal-packaged RFBG strain sensor attached was discretized with 134,952 hexahedral elements (SOLID185) due to the symmetry of both the geometry and loading. One half of the metal-packaged RFBG sensor was selected for mesh refinement and discretized with 125,952 hexahedral mesh elements, as shown in Figure 3a. As seen, finer mesh sizes were chosen at the location of the optical fiber and the sputtered and electroplated metallic layers. The surface to be connected on the specimen was defined as the target element (TARGE170), whilst the surface to be connected on the substrate was defined as the contact element (CONTA173). Structural loads are transferred from the surface of the specimen to the surface of the substrate via the spot-weld connection points. The boundary conditions and the load were applied to the one half of the structural model, as shown in Figure 3b.

The axial strains of the optical fiber were obtained from the 3-D FE simulation by obtaining an average strain for the nodes along the symmetry axis of the fiber and over 8-mm gauge length equivalent to the grating length of RFBG. Accordingly, the numerical results of the shifts in Bragg wavelength were calculated by substituting the axial and radial strains of the optical fiber into Equation (3).

## 4. Results and Discussion

### 4.1. Mechanical Strength Degradation of Silica Optical Fibers after Annnealing at High Temperatures

**Figure 4:**
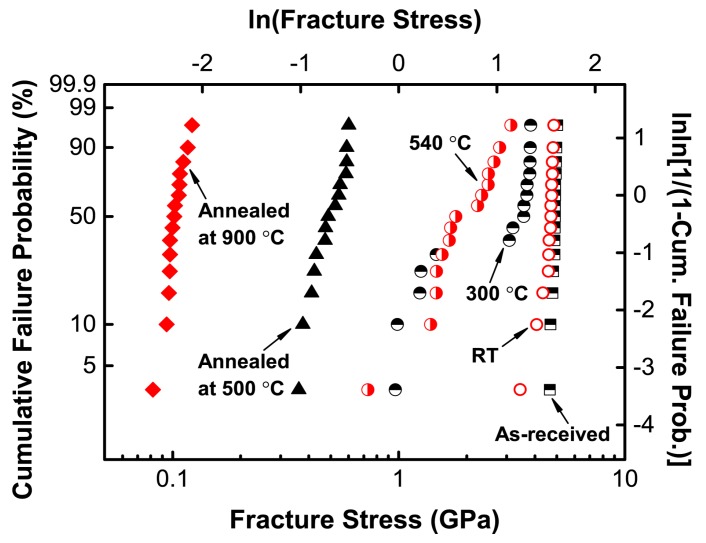
Weibull plot of the tensile strengths of the bare silica optical fibers annealed at 500 °C and 900 °C respectively and tested at room temperature (26 °C), compared with those of the bare fibers without annealing tested at room temperature (26 °C), 300 °C and 540 °C, as well as the as-received fibers tested at room temperature (26 °C) [26]. RT: room temperature

**Figure 5:**
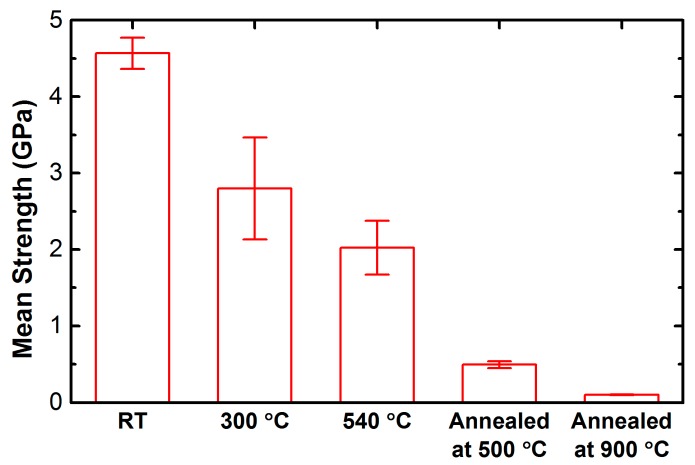
Mean tensile strengths of the bare silica optical fibers annealed at 500 °C and 900 °C respectively and tested at room temperature (26 °C), compared with those of the bare fibers without annealing tested at room temperature (26 °C), 300 °C and 540 °C [26].

Annealing at a high temperature is necessary for the fabrication of the RFBGs, although it usually leads to mechanical strength degradation of the silica optical fibers. It is thus essential to quantify the effects of annealing on the tensile strength of silica optical fibers. A summary of the results from the tensile tests of the silica optical fibers reported in our previous work [26] is shown in Figure 4. The fracture stress of all annealed fibers decreases precipitously after annealing at 500 °C and 900 °C. The mean tensile strengths determined by the fracture stresses collected from the tensile testing from 15 samples are shown in Figure 5. The results show that the annealing treatment leads to a significant reduction in the strength of the silica optical fibers after annealing at high temperatures. A particularly interesting result is that the strength of the annealed fibers is not only very much lower than that of the samples without annealing tested at room temperature, but it is also much lower than those of the samples tested at the temperatures of 300 °C and 540 °C, which is very similar to the behavior observed by others [24,27]. Figure 5 also shows that the higher temperature of annealing at 900 °C could lead to a larger reduction in strength of the fibers compared to those annealed at 500 °C. Silica fibers were found to become mechanically weaker after being annealed in air but the cause of such weakening was not well defined. Very recently, it was found that surface crystallization is probably responsible for the mechanical weakening observed in silica glass fiber surface after annealing at temperatures in excess of around 800 °C, while water diffusion-controlled virtual pitting of the glass surface is likely the source for the strength degradation at lower temperatures [27]. 

The results of the tensile tests indicate that the regeneration process of the RFBGs would considerably reduce the mechanical strength of silica optical fibers, even if careful preparations are integrated during the regeneration process. Moreover, the mechanical strength decreased significantly with the increased temperature of annealing at which the regeneration occurs. In order to extend the application of RFBG to high-temperature conditions, the annealing temperature needs to be carefully chosen as a function of the anticipated operating temperature and duration. To the best of the present authors’ knowledge, the annealing temperature of around 900 °C is the lowest temperature at which regeneration occurs for seed FBGs in H_2_-loaded SMF-28 fiber, whereas the annealing temperature of around 500 °C is the lowest temperature at which regeneration occurs for seed FBGs in PS1250/1500 photosensitive fiber. Accordingly, the mechanical strength of the PS1250/1500 fiber annealed at the lower temperature of 500 °C is expected to be higher than that of the SMF-28 fiber annealed at the higher temperature of 900 °C. Therefore, The H_2_-loaded PS1250/1500 fiber in which the RFBGs require a relatively lower regeneration temperature of 500 °C is a better choice as the material of sensing elements to develop high-temperature strain sensors.

### 4.2. Regeneration Characteristics of Regenerated Fiber Bragg Gratings

Figure 1 shows the evolution of the reflection peak power and the Bragg wavelength shift of a type-I FBG in PS1250/1500 fiber as a function of duration of the annealing. In addition, the temperature measured with the N-type thermocouple is also shown in Figure 1. The behavior of the gratings in H_2_-loaded PS1250/1500 fiber is significantly different in comparison with that of the gratings in H_2_-loaded SMF-28 fiber whose behavior has been described in our previous work [31]. The type-I FBG in H_2_-loaded PS1250/1500 fiber started to slowly decay after the temperature exceeded 150 °C (corresponding to ~200 °C for FBG in H_2_-loaded SMF-28 fiber). However, the decay of the FBG stopped at the temperature of ~300 °C, and subsequently the reflection peak power of the FBG started to increase slightly. Above ~430 °C, a rapid decrease in reflection peak power was observed up to the regeneration temperature of 500 °C for the FBG in H_2_-loaded PS1250/1500 fiber. The behavior of the FBGs in H_2_-loaded PS1250/1500 fiber exhibiting two regeneration temperature regimes is very similar to that of the FBGs in H_2_-loaded GF1B fibers reported by Polz et al. [35]. In contrast, for the FBGs in H_2_-loaded SMF28 fiber, only one regeneration regime above 900 °C was observed by the authors and other researchers [31,35]. Such behavior can be associated with the special type of fiber used. In order to achieve extremely high photosensitivity and to match mode field diameter (MFD) of the SMF-28 fiber, the core of PS1250/1500 fiber is co-doped with boron and germanium. The two regimes of regeneration may thus be related to the different structural relaxations that occur in the core and cladding where the dopant composition and concentrations differ, which has not been clarified to date. Further investigations are needed to clarify the relationship the two regeneration regimes with the specific fiber structure, dopant composition and concentrations.

**Figure 6:**
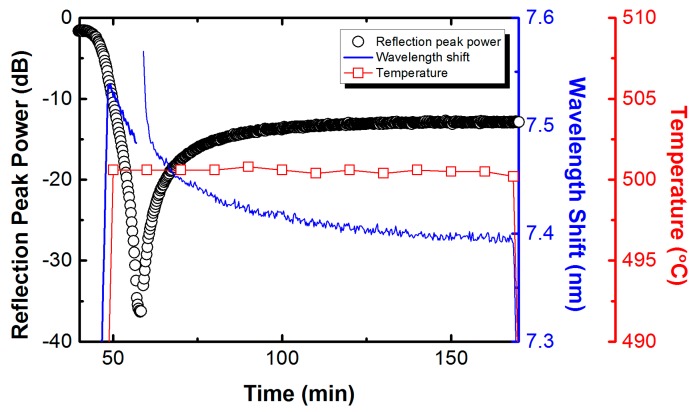
Evolution of the reflection peak power and the Bragg wavelength shift of the grating shown in Figure 1 during the isothermal annealing at 500 °C.

In addition to the decay of the grating, a shift of the Bragg wavelength has also been observed, as shown in Figure 6. The step of isothermal annealing at a temperature of 500 °C is zoomed. In this step, as the reflection peak power of the seed FBG fast decreases to the inflection point of complete erasure, the Bragg wavelength is abruptly shifted to shorter wavelengths, which indicates a strong decrease in both the refractive index modulation and average index change at this high temperature, and is typical behavior of thermal decay for a normal type-I FBG in PS1250/1500 fiber [36]. As the reflection peak power of the RFBG increases from the inflection point, its Bragg wavelength is much longer than that of its seed grating, consistent with the similar behavior reported in [37], which may be explained by the RFBG formed with changes (such as stress) at the core-cladding interface. After that, a significant negative shift in the Bragg wavelength of RFBG indicating a reduction in the average refractive index change is also observed until the wavelength gradually stabilizes at the end of the isothermal annealing of 500 °C. The trend of a negative shift in the Bragg wavelength for both seed grating and its regenerated grating is similar to that for the grating in H_2_-loaded SMF-28 fiber reported in our previous work [31] and also similar to that observed for regenerated type-IIA gratings [38]. However, it is inconsistent with other researchers’ observations that have shown a trend of a positive shift in the Bragg wavelength for the gratings in photosensitive fibers with different dopant composition and concentrations [35,37,39]. 

**Figure 7:**
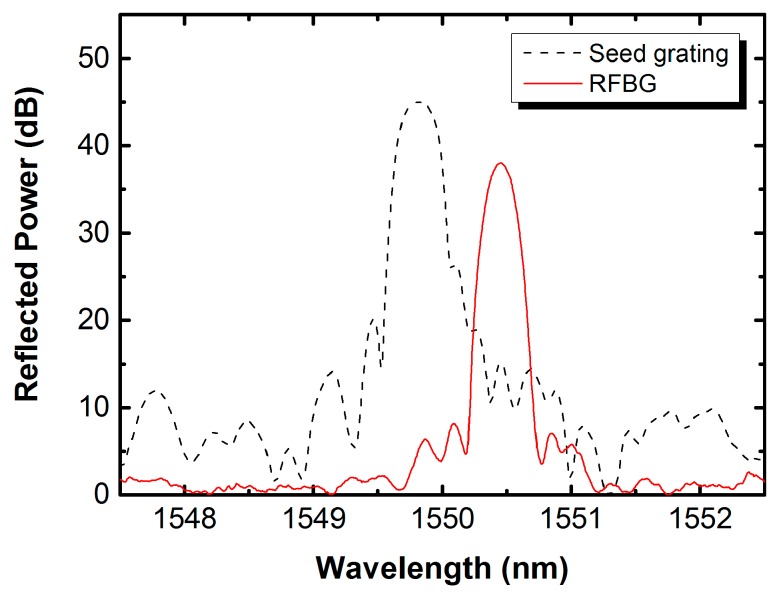
Reflection spectra of the typical type-I seed fiber Bragg grating (FBG) and its corresponding RFBG in H_2_-loaded PS1250/1500 fiber measured at room temperature (21 °C).

In sensing applications, it is desirable to have a good spectra profile and low reflectivity of sidelobes with respect to the main lobe of FBGs. Figure 7 shows a comparison of the reflection spectrum of the type-I seed grating inscribed in PS1250/1500 fiber to the spectrum of its corresponding RFBG observed at room temperature (21 °C). The spectrum of the seed grating presents a broad bandwidth and noticeable sidelobes, with a Bragg wavelength of 1549.813 nm, a 3 dB reflection bandwidth of ~0.281 nm, a sidelobe suppression ratio of ~15.5 dB, and a reflectivity of ~76.8%. In contrast to the seed grating, the RFBG provides a low reflectivity of ~9.6%, but has a good spectral profile with a better-defined peak (1550.450 nm), a lower 3 dB reflection bandwidth (~0.190 nm) and a larger reduction in sidelobe reflectivity (~−25.16 dB) compared to the main lobe. The spectral quality of the RFBG is significantly improved, arising from the high-temperature annealing treatment, which is sufficiently suitable for the built-in peak detection algorithm of most commercial FBG interrogators and is well applicable for multiplexing. The reason for this is that the thermally-activated defects induced by the high intensity pulses are annealed out, leaving a very smooth interface between the structurally altered and the unaffected region with the material. The reduction in overall reflectivity and bandwidth is directly proportional to the reduction of the refractive index modulation of the grating [5]. It is also observed that the Bragg wavelength of the RFBG is longer than that of its seed grating at room temperature, consistent with the behavior observed at 500 °C but not consistent with the behavior of the RFBG in H_2_-loaded SMF-28 fiber observed in our previous work [31]. The reason for the apparent spatial mismatch between the seed and regenerated gratings is that the regenerated grating is likely to have formed at the core-cladding interface boundary.

### 4.3. Strain Characteristics of Sensor Prototype

**Figure 8:**
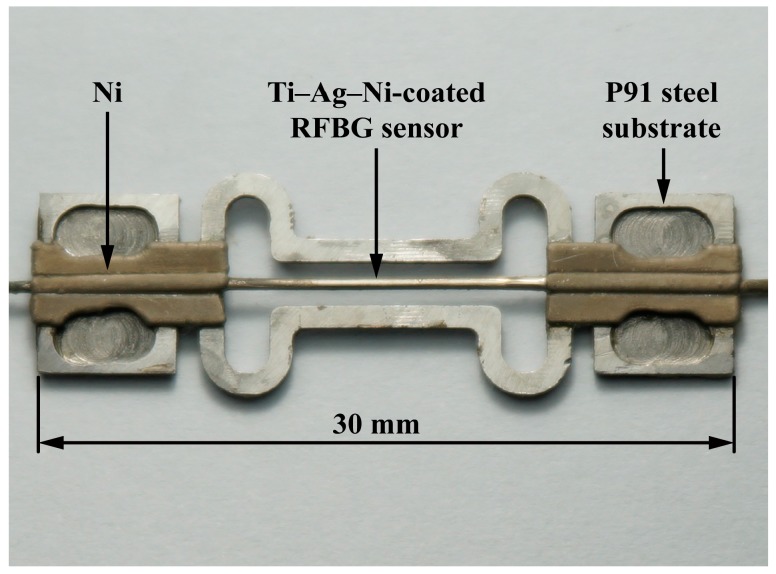
A laboratorial prototype of a metal-packaged strain sensor based on the RFBG in H_2_-loaded PS1250/1500 fiber.

Figure 8 shows a laboratorial prototype of a metal-packaged strain sensor based on use of the RFBG fabricated in H_2_-loaded PS1250/1500 fiber. The bare RFBG, sputter-coated with titanium and silver films with a total thickness of approximately 0.6 µm, and electroplated with nickel coating with a thickness of around 200 µm, is embedded in P91 steel substrate by nickel electroplating.

**Figure 9:**
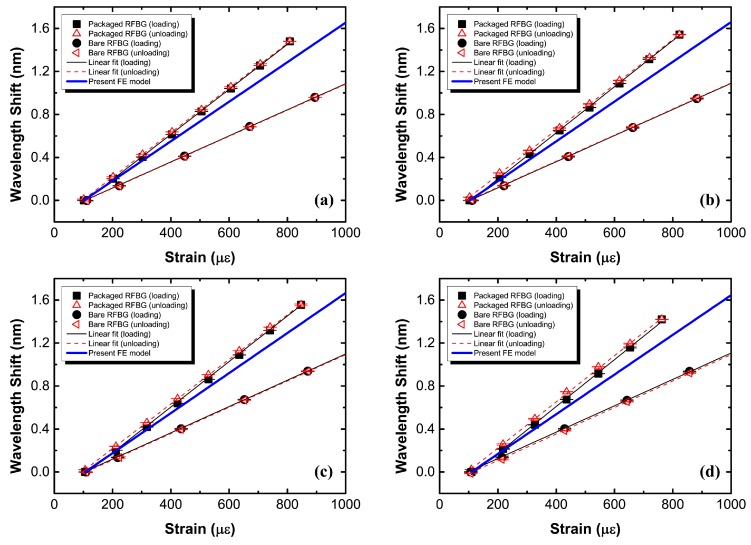

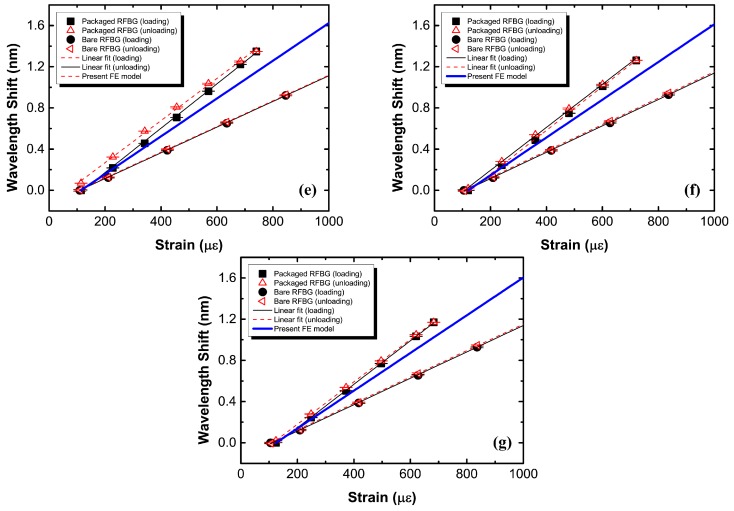
Shift in the Bragg wavelength as a function of strain obtained from tensile tests at constant temperatures of 26.5 °C (**a**); 100 °C (**b**); 200 °C (**c**); 300 °C (**d**); 400 °C (**e**); 500 °C (**f**) and 540 °C (**g**). FE: finite element.

To characterize the response to applied strain, the metal-packaged strain sensor based on the RFBG fabricated in H_2_-loaded PS1250/1500 fiber was mounted on the P91 steel specimen by spot welding. The specimen was loaded and unloaded at constant temperatures of room temperature (26.5 °C), 100 °C, 200 °C, 300 °C, 400 °C, 500 °C and 540 °C. Figure 9a–g illustrates the wavelength shifts of the metal-packaged RFBG strain sensor as a linear function of strains calculated from the applied forces, the cross-sectional area and Young’s modulus of the P91 steel specimen at the corresponding temperature (as listed in Table 1). The responses of the bare RFBG in H_2_-loaded PS1250/1500 fiber to the strains determined from the applied forces are also shown in Figure 9a–g for comparison. The observed shifts in Bragg wavelength and strains show linearity for both metal-packaged and bare RFBG sensors, with the adjusted coefficients of determination (adj R^2^) higher than 0.999 for the former and 0.9999 for latter. The metal-packaged sensor preserves its linear behavior implying a good interfacial integrity between every two layers and elastic deformations in each material. The strain sensitivities derived from the slope of the straight lines in Figure 9a–g are 2.10, 2.15, 2.12, 2.17, 2.15, 2.12 and 2.11 pm µε^−1^ for the metal-packaged RFBG sensor under loading at constant temperatures of room temperature (26.5 °C), 100 °C, 200 °C, 300 °C 400 °C, 500 °C and 540 °C, respectively, which is slightly higher than those of 2.08, 2.10, 2.08, 2.15, 2.04, 2.09 and 2.06 pm µε^−1^ under unloading, as elastic hysteresis occurs in the relatively flexible structural substrate. In addition, at corresponding test temperatures, the values are ~30% higher than the values of the metal-packaged strain sensor fabricated based on the RFBG in H_2_-loaded SMF-28 fiber reported in our previous work [17]. This could be mainly attributed to the differences in geometrical dimensions (the thickness of the electroplated nickel coating, the depth of the fiber embedded into the substrate, etc.) of the packaged structure fabricated manually, and inaccuracy in the material parameters (Young’s modulus, etc.) used to calculate the strains to which the steel specimen is subjected. The thinner coating of electroplated nickel and the deeper location of the fiber embedded into the substrate would result in the higher strain sensitivity of the sensors. The value of Young’s modulus for P91 steel used to calculate the strains to which the specimen is subjected may be slightly greater than the true value. Accordingly, the calculated strains are smaller than the true strains to which the P91 steel specimen is subjected, leading to the higher sensitivity that is the ratio of the wavelength shift to the calculated strain.

The experimental results of the tensile tests performed on the bare RFBG in H_2_-loaded PS1250/1500 fiber are plotted in Figure 9a–g for comparison with those of the metal-packaged RFBG strain sensor. We can obtain the strain sensitivities of 1.21, 1.22, 1.22, 1.23, 1.25 and 1.28 pm µε^−1^ for the bare RFBG sensor under loading at constant temperatures of room temperature (21 °C), 100 °C, 200 °C, 300 °C, 400 °C and 500 °C respectively, in contrast to those of 1.21, 1.21, 1.22, 1.23, 1.25 and 1.28 pm µε^−1^ under unloading. They are slightly higher than values of the bare RFBG in H_2_-loaded SMF-28 fiber reported in our previous work [17]. The strain sensitivity of the metal-packaged RFBG sensor is ~70% higher than that of the bare RFBG sensor due to the flexible structure of the substrate specially designed to enhance the strain sensitivity. An approximation of the nominal gauge length (see Figure 8) of the metal-packaged RFBG strain sensor in measurement axis is the distance of between the weld spots. The true gauge length of the strain sensitive section of the sensor is the length of metal-coated fiber containing the RFBG, measured by the inside of the flexible structure, which is shorter than the nominal gauge length. The structural deformations to be measured over the nominal gauge length mainly result in elongation over the true gauge length rather than elongation over the nominal gauge length, since the rigidity over the section of the substrate without the flexible structure is much greater than that over the section with the flexible structure. Here, the strain sensitivity of sensor is the ratio between the shift in Bragg wavelength of the strain sensor induced by the average unit elongation over the true gauge length and the nominal strain determined from the applied force, cross-sectional area and Young’s modulus of the structure to be measured, corresponding to the average unit elongation over the nominal gauge length. As a result, the strain sensitivity of the metal-packaged sensor is higher than that of the bare RFBG sensor due to the flexible structure.

**Figure 10:**
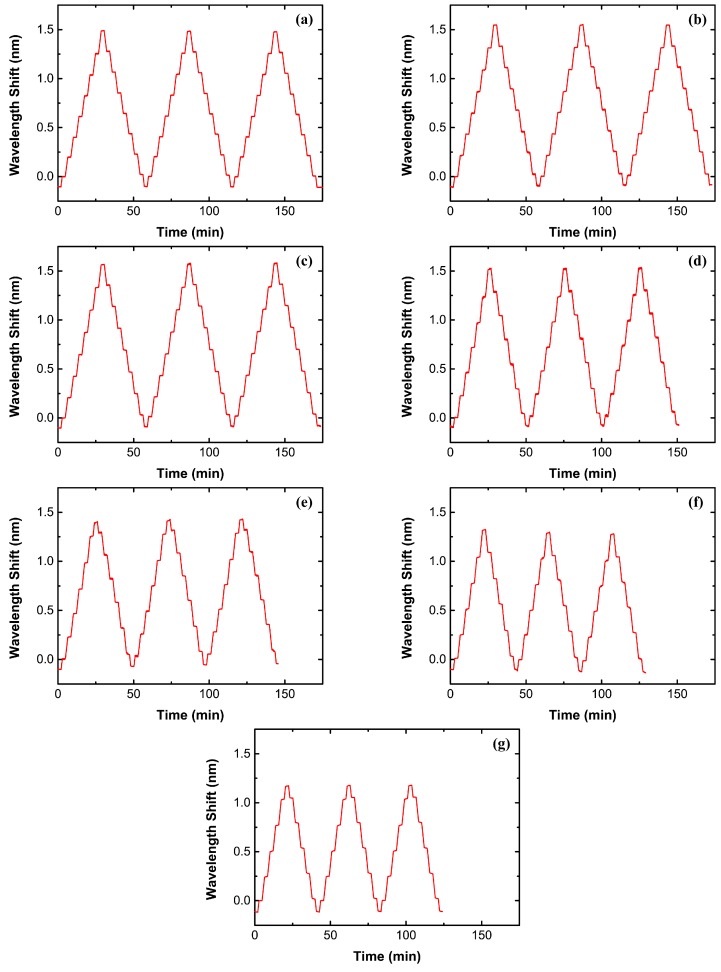
Temporal evolution of the wavelength shift during the mechanical loading cycles for the metal-packaged strain sensor based on the RFBG in H_2_-loaded PS1250/1500 fiber at constant temperatures of 26.5 °C (**a**); 100 °C (**b**); 200 °C (**c**); 300 °C (**d**); 400 °C (**e**); 500 °C (**f**) and 540 °C (**g**).

**Figure 11:**
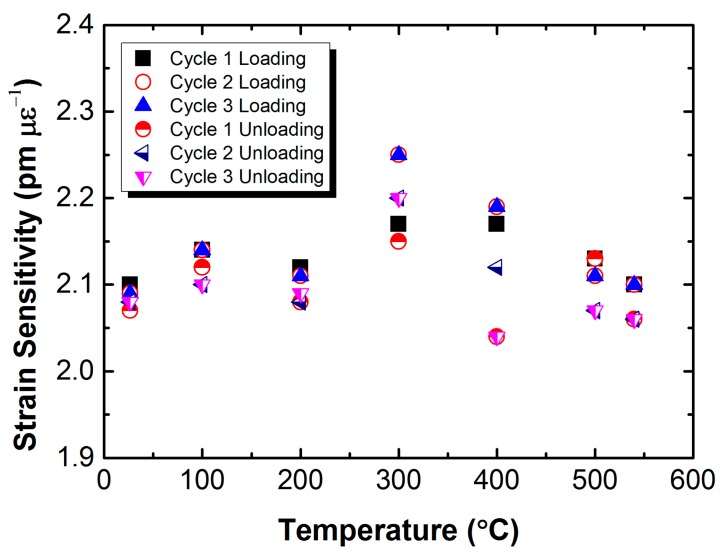
Strain sensitivity of the metal-packaged strain sensor based on the RFBG in H_2_-loaded PS1250/1500 fiber obtained from mechanical load-cycling tests as a function of temperature.

For sensor applications, the Bragg wavelength of a RFBG-based strain sensor must be stable and repeatable when subjected to loading at high temperatures. Accordingly, the tensile tests were conducted at the same test temperatures for three times to determine its stability and repeatability. Figure 10a–g shows the temporal evolution of the shift in Bragg wavelength during the mechanical-loading cycles for the sensor prototype at constant temperatures of 26.5 °C, 100 °C, 200 °C, 300 °C, 400 °C, 500 °C and 540 °C, with the same scale in both the wavelength shift and time. It is observed that the sensor prototype presents no obvious drift in its Bragg wavelength. Each shift in Bragg wavelength of the sensor prototype is calculated by averaging the shifts measured as the load is held constant within 2 min to determine its strain sensitivity. These values obtained from the mechanical loading-cycling tests agree well with one another, as shown in Figure 11. Slight fluctuations in the wavelength shift of the sensor prototype are related to temperature disturbance at temperatures of 300 °C and 400 °C. These results thus highlight the importance to compensate the temperature effect to enhance the accuracy. The experimental results demonstrate that the metal-packaged strain sensor based on the RFBG fabricated in H_2_-loaded PS1250/1500 fiber has good stability and repeatability. It has also been proven that the interfacial bonding between the optical fiber and the titanium layer is strong, as well as the nickel layer and the P91 steel substrate.

### 4.4. Numerical Results

The numerical results estimated by implementing the developed 3-D FE model of the metal-packaged RFBG strain sensor are also shown in Figure 9a–g, for comparison with the experimental results. The shifts in the Bragg wavelength calculated by substituting the axial and radial strains of optical fiber obtained from the FE simulation into Equation (3), and strain values determined from the forces applied to the specimen are linear. The numerical strain sensitivities of 1.84, 1.85, 1.86, 1.85, 1.83, 1.83, and 1.83 pm με^−1^ are derived from the slope of the solid straight blue lines shown in Figure 9a–g, respectively. Comparisons of the experimental results and the numerical results show a satisfactory agreement with a relative error less than 15.7%, which may be primarily attributed to the inaccuracy in the material parameters (particularly the P91 steel) use in 3-D FE model and the errors in the measurement of structural dimensions. 

**Figure 12:**
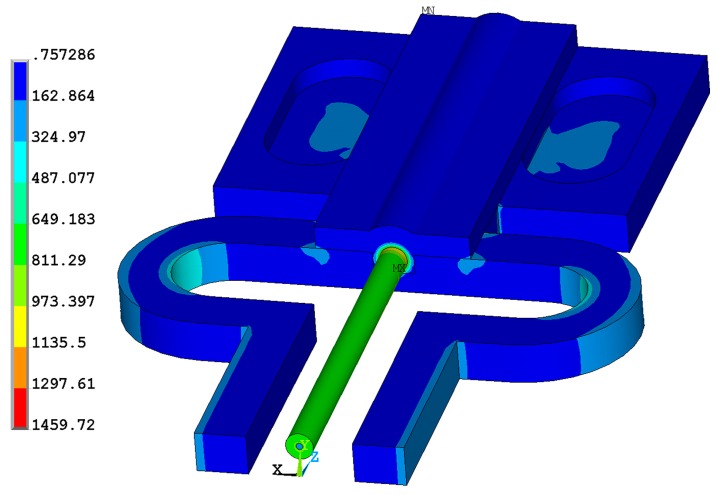
Von Mises stress distribution in the metal-packaged RFBG strain sensor at a strain of 0.26% applied to the test specimen at room temperature.

To avoid undesirable plastic deformation of the P91 steel specimen, the specimen was only tested up to ~0.08% strain, corresponding to ~0.12% strain in the RFBG obtained from the FE simulation. For further loading, the verified linear trend may be maintained up to the strain limit of the bare RFBG (i.e., ~0.4% at 4 N as discussed in previous sections, corresponding to ~0.26% strain in the specimen) which restricts the strain measurement range of the sensor. However, this is also largely dependent on the behavior of the substrate. At a strain of 0.26%, the von Mises stresses occurring in the substrate are determined from the FE modeling at the room temperature, as shown in Figure 12. Assuming the spot welds with sufficient strength to transfer the structural loads from the specimen to the sensor, the maximum von Mises stress occurring in the nickel layer far exceeds the yield strength of 59.0 MPa [32] in addition to yielding occurring in the region of the spot welds, which confirms that the strain measurement range of the sensor is limited not only by the strain range of the RFBG, but also by the strength of the metallic packaging materials and the spot welds.

## 5. Conclusions

In this paper, the results of the development and characterization of a laboratorial prototype of a metal-packaged strain sensor based on the use of an RFBG fabricated in H_2_-loaded B–Ge co-doped photosensitive fiber have been reported. Some conclusions have been drawn from the investigation as follows:

1. Regenerated fiber Bragg gratings fabricated in fibers (e.g., B–Ge co-doped photosensitive fiber) which require a relatively lower regeneration temperature (e.g., regeneration temperature of 500 °C) are preferred as the sensing elements to develop high-temperature strain sensors. This takes into consideration that the regeneration process considerably reduces the mechanical strength of the silica optical fibers for which degradation becomes more severe after annealing at higher regeneration temperatures even if careful preparations are integrated during the regeneration process.
2. The metal-packaged strain sensor prototype based on the use of the RFBG fabricated in H_2_-loaded PS1250/1500 fiber exhibits good linearity, stability and repeatability when exposed to constant high temperatures up to 540 °C, which is higher than the upper operating temperature limit of 400 °C for the strain sensor based on the RFBG in H_2_-loaded SMF-28 fiber reported in our previous work [17]. Strain sensitivity of the metal-packaged sensor is ~70% higher than that of the corresponding bare RFBG due to the support of flexible structure of the metallic substrate.
3. Anomalous decay behavior of exhibiting two regeneration regimes has been found for the FBGs written in H_2_-loaded PS1250/1500 fiber, with reflectivity showing a small but clear increase at temperatures between ~300 °C and ~430 °C, interpreted as a first regeneration regime. Above ~430 °C, the gratings started to decay up to 500 °C where the FBGs regenerated, which is interpreted as a second regeneration regime. This is similar to the behavior of the FBGs in H_2_-loaded GF1B photosensitive fiber observed by Polz et al. [35]. In contrast to that, for the FBGs in H_2_-loaded SMF28 fiber, only one regeneration regime above 900 °C was observed. The two regimes of regeneration may be related to the different structural relaxations that occur in the core and cladding where the dopant composition and concentrations differ.
4. Comparisons of the experimental results and the numerical results of strain sensitivity for the metal-packaged strain sensor prototype show a satisfactory agreement with a relative error less than 15.7% in the range of the test temperature, which may be primarily attributed to the inaccuracy in the material parameters, particularly of the P91 steel, used in 3-D FE model and the errors in the measurement of structural dimensions. The FE simulation also shows the operational strain range of the sensor is limited not only by the strain measurement range of the RFBG, but also by the strength of the metallic packaging materials and the spot welds.

The metal-packaged strain sensors using silica optical fibers provide great potential for strain measurements in high-temperature environments in ways that were not possible before. However, the strength degradation of silica optical fibers after annealing at high temperatures may also impedes the practical utility of the RFBG-based strain sensors. The mechanical integrity and packaging remain as the key challenges. In future work, it is crucial to improve material systems to meet higher temperature challenges.

## Figures and Tables

**Table 1:** Material parameters for three-dimensional (3-D) finite element (FE) analysis [32,33,34].

Parameter	Temperature (°C)						
	26.5	100	200	300	400	500	540
*E*_fiber_ (GPa)	72.9	73.8	74.95	76.04	77.036	77.936	78.28
ν_fiber_	0.17	0.17	0.17	0.17	0.17	0.17	0.17
*E*_titanium_ (GPa)	116	112	106	100	95	89	87
ν_titanium_	0.34	0.34	0.34	0.34	0.34	0.34	0.34
*E*_silver_ (GPa)	76	71	65	59	52	46	43.5
ν_silver_	0.37	0.37	0.37	0.37	0.37	0.37	0.37
*E*_nickel_ (GPa)	217	201	180	194	204	195	191
ν_nickel_	0.31	0.31	0.31	0.31	0.31	0.31	0.31
*E*_P91_ (GPa)	220	216	210	204	195	185	179
ν_P91_	0.29	0.29	0.29	0.30	0.29	0.30	0.29
*µ*_P91_	0.15	0.15	0.15	0.15	0.15	0.15	0.15
